# Ductus Venosus Agenesis in Monochorionic Twin Pregnancies Complicated by Fetal Growth Restriction: When to Deliver?

**DOI:** 10.3390/diagnostics14192147

**Published:** 2024-09-26

**Authors:** Eleonora Torcia, Alessandra Familiari, Elvira Passananti, Giulia di Marco, Federica Romanzi, Mariarita Trapani, Daniela Visconti, Antonio Lanzone, Elisa Bevilacqua

**Affiliations:** 1 Department of Women and Child Health, Women Health Area, Fondazione Policlinico Universitario Agostino Gemelli, IRCCS, 00168 Rome, Italy; eleonora.torcia@gmail.com (E.T.); alessandra.familiari@policlinicogemelli.it (A.F.); elvira.passananti@gmail.com (E.P.); giulia.dimarco1102@gmail.com (G.d.M.); federica.romanzi@gmail.com (F.R.); daniela.visconti@policlinicogemelli.it (D.V.); antonio.lanzone@policlinicogemelli.it (A.L.); 2 Unit of Obstetrics and Gynecology, Università Cattolica del Sacro Cuore, 00168 Rome, Italy; mariarita.trapani@gmail.com

**Keywords:** fetal medicine, multiple pregnancy, prenatal diagnosis, ultrasound, high-risk pregnancy, ductus venosus agenesis

## Abstract

**Introduction:** The prevalence of ductus venosus agenesis (ADV) in singleton pregnancies ranges from 0.04% to 0.15%, while its prevalence in twins remains largely unknown. To our knowledge, in the literature, there is only a single case report of a monochorionic diamniotic (MCDA) pregnancy complicated by ADV. Fetuses with ADV are at increased risk for congenital cardiac disease, heart failure, and fetal growth restriction (FGR). Consequently, these pregnancies have a heightened risk of experiencing an adverse outcome, like stillbirth and neonatal or infant death. Closer antenatal monitoring is warranted when ADV is suspected. Currently, there are no guidelines regarding the standard of care in cases of ADV and no recommendations for the timing of delivery in either singleton or twin pregnancies. **Cases:** This study aims to provide a comprehensive overview of the management of twin pregnancies complicated by ADV, featuring two cases of MC twins with concurrent sFGR and ADV in one twin. **Discussion:** These pregnancies experienced completely different outcomes, underscoring the necessity for personalized management tailored to the specific risk factors present in each pregnancy. Typically, in MCDA pregnancies with severe sFGR (type II and III), delivery represents the most reasonable option when venous Doppler abnormalities are identified. However, the absence of the DV complicates the management and the process of decision-making regarding the timing of delivery in cases of sFGR and ADV. We emphasize that effective decision-making should be guided by the presence of additional risk factors, including velamentous insertion, significant estimated fetal weight discordance, and progressive deterioration of the Doppler over time. **Conclusions:** Our experience suggests that these factors are strongly correlated with poorer outcomes. Given this context, could it be acceptable, in the case of MC pregnancy complicated by severe sFGR and ADV, with worsening findings and additional risk factors (e.g., velamentous insertion, severe birth weight discrepancy), to anticipate the time of delivery starting from 30 weeks of gestational age?

## 1. Introduction

The ductus venosus (DV) is a small vessel that diverts oxygenated blood from the umbilical vein (UV) to the inferior vena cava (IVC) and the right atrium (RA) of the fetal heart, effectively bypassing the fetal liver. It constitutes one of the three physiological shunts in the fetal circulation and plays a crucial role in fetal adaptation to hypoxic conditions [1,2]. Under normal circumstances, the DV diverts 25% of umbilical venous flow toward the RA in a high-velocity stream, while 55% of the flow is directed to the left liver lobe and 20% to the right liver lobe [3]. This distribution of umbilical venous blood has significant implications, particularly for the glucose–IGF–insulin-mediated growth axis of the fetus, which is a key determinant of longitudinal fetal growth [3,4,5,6]. Moreover, high-velocity blood flow in the DV modulates the hemodynamic mechanisms for intracardiac partitioning of the blood streams, so a decline in the velocity of nutrient-rich blood directed toward the left atrium could lead to a reduction in oxygen for the myocardium and fetal brain [4,7]. Finally, proper umbilical flow through the DV is critical for the establishment of the intrahepatic portal venous system (IHPVS). Anatomical variations during fetal development of specific vascular anastomoses between the portal, umbilical, and hepatic–systemic venous systems can lead to abnormal circulation and, in some cases, to the absence of DV, known as ductus venosus agenesis (ADV) [8]. The reported prevalence of ADV varies in the literature, ranging from 0.04% and 0.6% [9]. In a recent retrospective study involving 8304 singleton pregnancies, the prevalence of ADV was 0.15%, with 9 out of 13 (69%) of these cases exhibiting UV drainage into the portal system and a favorable outcome. In contrast, in 4 out of 13 (31%) fetuses with extrahepatic drainage of the UV, increased nuchal translucency (NT), and structural or chromosomal anomalies were detected [10]. According to Piemonti et al., fetuses with extrahepatic UV drainage are at greater risk of congenital heart disease and FGR, due to aberrant vascular anatomy of the extrahepatic shunt, causing an important cardiac rebound. Consequently, these fetuses have a higher probability of experiencing an adverse outcome, including stillbirth or neonatal or infant death, warranting closer antenatal surveillance in suspected cases of ADV [6].

While arterial Doppler evaluation provides important details about placental function and its impact on fetal circulation, venous Doppler evaluation is critical for a comprehensive assessment of fetal well-being by quantifying fetal cardiovascular compromise [11]. DV is perceived to be the optimal tool for predicting fetal acidemia and is widely regarded as the most crucial parameter for determining the timing of delivery in cases of preterm FGR [12]. In singleton pregnancies complicated by early-onset FGR, indeed, Doppler velocimetry of the DV plays a central role in diagnosis, surveillance, and management. In these complex cases, several studies have shown that delivery decisions based on DV waveform deterioration optimize neonatal outcomes [13,14]. However, in pregnancies complicated by ADV, the inability to utilize this parameter for delivery timing presents a significant challenge, as evidence-based guidelines for managing such cases are lacking. To our knowledge, the incidence of ADV in twin pregnancies remains unknown. In monochorionic (MC) pregnancies, only one case of ADV has been reported in the literature [15].

It is well known that MC twin pregnancy is associated with an increased rate of adverse perinatal outcomes and, in particular, with well-defined placenta-related complications such as twin-to-twin transfusion syndrome (TTTS), selective FGR (sFGR), and twin anemia–polycythemia sequence (TAPS) [16].

sFGR occurs in approximately 10–15% of cases and represents a clinical challenge, as the risk of stillbirth in a growth-restricted fetus must be balanced against the risk of prematurity for both twins [16,17]. To standardize the diagnostic features of sFGR in twin pregnancies, a Delphi consensus established specific guidelines for diagnosing sFGR in MC pregnancies [18]. The condition can be further classified by the pattern of end-diastolic flow (EDF) in the umbilical artery (UA). In type I, the UA Doppler waveform has positive EDF. In type II, there is absent or reversed EDF (AREDF). In type III, there is a cyclical/intermittent pattern of AREDF [19]. According to international guidelines for sFGR, fetal growth should be assessed at least every 2 weeks and fetal Doppler (UA and middle cerebral artery, MCA) at least weekly. If the UA Doppler is abnormal, an assessment of the DV blood flow should be carried out. The timing of delivery should be decided based on the evaluation of fetal well-being, interval growth, biophysical profile, DV waveform, and/or computerized cardiotocography (cCTG), when available [20,21].

In cases of ADV, as with singleton pregnancies, no specific guidelines for standard care exist. This report describes the largest case series to date, including two cases of MC twin pregnancies complicated by sFGR and ADV of one twin, with known obstetric and neonatal outcomes.

## 2. Case 1

**Figure 1:**
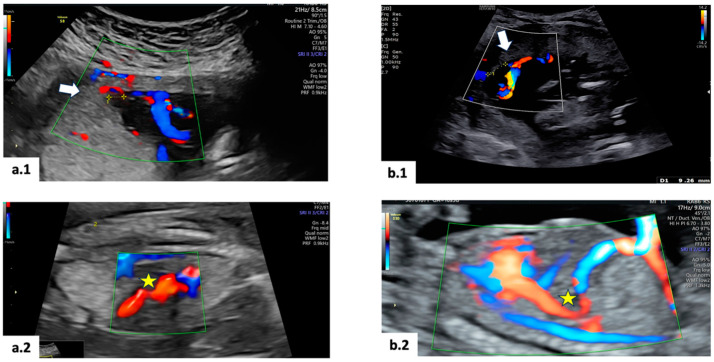
Main ultrasound characteristics: (**a.1**) velamentous insertion highlighted by ➭ and (**a.2**) ADV and UV drainage in the IVC highlighted by ★, in the first case; (**b.1**) marginal insertion highlighted by ➭ and (**b.2**) ADV and UV drainage in the IVC highlighted by ★, in the second case.

**Figure 2:**
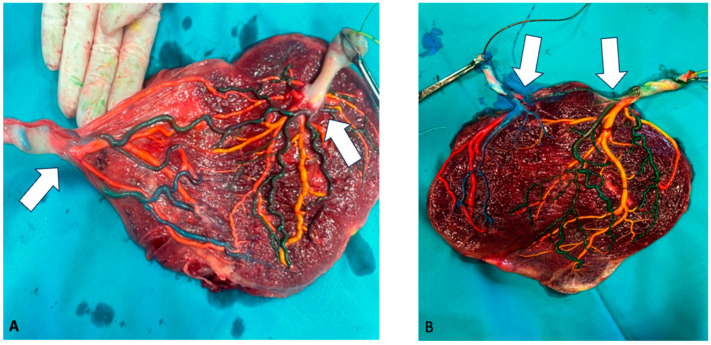
Placenta after color-dye injection (for twin I: yellow vein, green artery; for twin II: red vein, blue artery). (**A**) Discordant umbilical cords’ insertion (white arrows) and placental anastomosis in the first case, after color-dye injection technique. (**B**) Concordant umbilical cords’ insertion (white arrows) and placental anastomosis in the second case, after color-dye injection technique.

A 36-year-old Caucasian woman, primigravida, with an MCDA pregnancy, was followed at the Twin Clinic of Agostino Gemelli University Policlinic IRCCS in Rome, Italy. The only remarkable issue in her medical history was type II diabetes. The pregnancy was achieved via assisted reproductive technologies (ARTs), with both sperm and oocyte donation. During the first-trimester screening scan at 13 weeks and 4 days of gestational age (GA), the NT measurement was within the normal range for both twins, but ADV was suspected in fetus 2. The insertion sites of the umbilical cord were discordant (Figure 1): central for the first twin and velamentous for the second one. A fibroid measuring 66 × 72 mm was identified in the posterior uterine wall beneath the placenta. A diagnosis of type II sFGR with no signs of superimposed TTTS was given at 16 + 5 weeks of GA. ADV was confirmed, with UV drainage into the IVC. Figure 1 illustrates the primary ultrasound findings. Given the early GA at diagnosis, different management options, including intensive ultrasound monitoring versus prenatal interventions (such as cord occlusion or laser coagulation of placental anastomoses), along with associated risks, were thoroughly discussed. The couple decided on an intensive ultrasound follow-up. At 28 weeks + 1 day, the sFGR twin showed absent EDF in the UA, and the estimated fetal weight (EFW) discordance raised to 30%. The patient was hospitalized for daily cCTG and fetal Doppler ultrasound every 2–3 days. Antenatal corticosteroids were administered. A multidisciplinary case discussion concluded that delivery should be scheduled at 32 weeks in the absence of clinical or ultrasound deterioration. However, in the event of worsening Doppler ultrasound findings and/or cCTG abnormalities, immediate delivery would be performed. Ultrasound evaluation and short-term variability (STV) at cCTG were always unremarkable until 30 weeks GA, when intrauterine demise of the second twin with sFGR was diagnosed. An emergency cesarean section (CS) was performed due to cCTG abnormalities of the survived twin. At birth, the first twin weighed 1330 g, with an APGAR score of 6/8/10. The demised twin weighed 990 g. The surviving twin was admitted into the neonatal intensive care unit (NICU), requiring continuous positive airway pressure (CPAP) ventilation. Subsequently, he developed neonatal jaundice treated with phototherapy. Brain ultrasound at 7 days of life revealed a grade IV periventricular leukomalacia (PVL). A cerebral MRI at 7 weeks of life confirmed severe PVL, characterized by the absence of cerebral parenchyma in parieto-temporal lobes bilaterally and reduced cortical gyration. The infant was discharged at 2 months and 6 days of life with neuromotor impairment and limited spontaneous mobility. Post-delivery, the placenta was stored at 4 °C and examined by color-dye injection. This technique highlighted the presence of anastomosis on the chorionic plate, which was classified as follows: 1 arterio-arterial (AA), 4 arterio-venous (AV), 3 veno-arterial (VA), and 1 veno-venous (VV) (Figure 2).

## 3. Case 2

A 30-year-old Caucasian woman, primigravida, with an MCDA pregnancy, was followed in the Twin Clinic of Agostino Gemelli University Policlinic IRCCS in Rome, Italy. Her medical history was unremarkable. The first scan performed at 13 + 6 weeks GA revealed a discrepancy in the NT (3 vs. 1.10 mm) and in the crown–rump length (CRL) (74 vs 83 mm) between twins. Additionally, ADV was suspected in the first fetus. The umbilical cord insertion sites were concordant for both twins, with marginal cord insertion. Chorionic villus sampling (CVS) was performed externally at 11 + 5 weeks GA, revealing a normal karyotype, 46 XX. A diagnosis of type II sFGR with no signs of superimposed TTTS was made at 15 + 6 weeks GA. ADV was confirmed, with UV drainage into the IVC. Figure 1 illustrates the main ultrasound information. As for the case described above, after discussing the various management options and associated risks, the couple opted for intensive ultrasound follow-up. During the surveillance, the ultrasound findings remained stable, with partial improvement of the pulsatility index (PI) in the UA for the first twin, with EDF consistently present from 20 weeks to 32 + 6 weeks GA. The estimated fetal weight (EFW) discordance remained at 22.9%, with no additional complications. Weekly monitoring was performed until 34 weeks GA, when an elective cesarean section (CS) was scheduled following the administration of corticosteroids for lung maturation. At delivery, the first twin had an APGAR score of 9/9/10 and weighed 1470 g, while the second twin had an APGAR score of 9/10/10 and a weight of 1900 g. Both twins were admitted to the NICU due to neonatal respiratory distress syndrome (RDS) requiring CPAP ventilation and neonatal jaundice. A brain ultrasound performed in the first week of life yielded normal results for both twins. They were discharged 17 days after birth in a stable clinical condition. Post-delivery, the placenta was stored at 4 °C and analyzed by color-dye injection that highlighted the presence of anastomosis on the chorionic plate that was classified as follows: 1 AA, 3 AV, 4 VA, and 0 VV (Figure 2).

## 4. Discussion

To the best of our knowledge, this is the largest case series describing the management of MCDA pregnancies complicated by ADV. Only one previous case report, published in 1996 by Shih et al., described an MC pregnancy, complicated by TTTS, sFGR, and ADV [15]. In this case, the “recipient” twin had a diagnosis of ADV, with the UV draining directly into the infracardiac portion of the IVC. This anomaly was also confirmed at the post-mortem examination, following the intrauterine demise of both twins at 33 weeks of gestation. The authors hypothesized that the hemodynamic consequences of the ADV may have contributed to volume overload in one twin, potentially mimicking the clinical features of TTTS [15]. This scenario was partially observed in our first case, where an imbalance of amniotic fluid (deepest vertical pocket (DVP) of 62 mm vs. 26 mm) was detected at 16 + 5 weeks GA. However, in our cases, the fetuses affected by ADV developed an sFGR without evidence of superimposed TTTS. Shih et al. described vascular anastomosis in their case, though they did not provide details on the insertion of the umbilical cords [15]. According to Bonanni et al., there is a strong association between velamentous cord insertions and adverse neonatal outcomes, as well as between twin birth weight discordance and discordant umbilical cord insertion sites (comprising central, paracentral, eccentric, and velamentous insertions) [22]. In the first case described in this series, which had the worst neonatal outcome, the twin affected by ADV had a velamentous cord insertion with a discordant insertion site, whereas in the second case, the umbilical cord insertions were concordant. This finding emphasizes the importance of assessing cord insertion sites in predicting perinatal complications for MC twin pregnancies, especially when other risk factors, such as ADV and sFGR, are present. The first-trimester screening at 11–13+6 weeks and the routine anomaly scan at 20–22 weeks were performed according to national and international guidelines [20,23]. According to Garcia-Delgado et al., the ADV or an abnormal umbilical vein course should lead to a detailed anatomical evaluation to assess for associated structural anomalies, as this condition is significantly linked to both cardiac and extracardiac malformations. Fetuses with ADV, especially those with extrahepatic drainage of the UV, should be monitored for signs of congestive heart failure and hydrops, even in the absence of associated cardiac defects [24]. None of the fetuses affected by ADV in our case series exhibited morphological anomalies, nor did they develop hydrops or signs of heart failure. Few studies have explored the obstetric and neonatal outcomes of singleton pregnancies with ADV [25]. The prognosis appears to depend on the pattern of the abnormal venous circulation, as well as the presence of associated malformations and chromosomal abnormalities [26]. In both our cases, the UV drained in the abdominal part of the IVC, which likely prevented the right atrial overload described by Shih et al. [15]. Careful serial sonographic evaluations are crucial, as in the case of severe fetal compromise, early delivery may be necessary not only to prevent stillbirth in the affected fetus but also to mitigate potential complications for the healthy co-twin [27]. The TRUFFLE Study demonstrated that in singleton pregnancies with severe early-onset FGR, the timing of delivery based on DV Doppler measurement, in conjunction with cCTG safety-net criteria, improves long-term neurodevelopmental outcomes at two years in surviving infants [13,14]. In MCDA pregnancies, the management of sFGR is even more challenging, given the higher risk of stillbirth, the potential consequences of preterm delivery for both fetuses, and the unpredictable occurrence of complications related to placental anastomoses. For these reasons, particularly for the first case, we based our clinical management not only on the deterioration of fetal Doppler parameters but also on the cCTG interpretation.

## 5. Conclusions

In this study, we describe two MCDA pregnancies at extremely high risk due to additional risk factors, such as sFGR with ADV in one twin. The first case was further complicated by velamentous cord insertion, while the second case presented a discrepancy in CRL and NT in the first trimester. Despite these similarities, the outcomes were markedly different: the first case ended with the most unfavorable outcome (one fetus with in utero demise and one child with severe morbidity), whereas the second one culminated in the best desired possible outcome (both twins being born healthy and near term). A comparative analysis reveals critical differences; the first case exhibited velamentous insertion and deterioration of the Doppler findings over time, ultimately necessitating hospitalization. In contrast, the second case demonstrated stable sFGR with an improvement in Doppler findings, never requiring intensive inpatient surveillance.

The optimal management of sFGR in MC pregnancies remains exceedingly challenging and poorly defined. Recent guidelines on twin pregnancy have also highlighted this lack of evidence [21]. Despite advances in antenatal care, there is still a wide variation in the diagnostic criteria, management, and, more importantly, GA threshold for delivery. Management should be personalized, taking into account GA at diagnosis, the degree of fetal weight discordance, and the severity of Doppler abnormalities [28]. Typically, in MCDA pregnancies with severe sFGR (type II and III), delivery when venous Doppler abnormalities occur represents the most reasonable option [28]. According to existing guidelines and recommendations, in cases of sFGR, delivery time should be decided according to Doppler assessment of the UA and DV. If DV is abnormal (absent a-wave), delivery is suggested starting from 26 weeks, while if there is absent or reverse EDF in UA and DV remains normal, delivery should be contemplated starting from 32 weeks [20,29]. According to our institutional protocol in cases of sFGR of type II and III (in fetuses without ADV) with a pulsatility index (PI) value of DV greater than 95°, an earlier delivery starting from 30 weeks may be considered.

We hypothesize that, in cases of sFGR of type II and III in twins with ADV compounded by additional risk factors (e.g., velamentous insertion, severe birth weight discrepancy), delivery could be considered starting from 30 weeks. Further studies are needed to understand how to properly stratify pregnancies complicated by ADV. Velamentous cord insertion remains a significant additional risk factor strongly associated with adverse outcomes, further reinforcing the need to incorporate it into the management of these pregnancies. It is urgent and necessary for the scientific community to focus future research efforts on large multicenter prospective trials to determine the optimal management of MC pregnancies complicated by sFGR, taking into account the GA at diagnosis, type and severity of UA Doppler pattern, degree of weight discordance, and presence of additional risk factors.

## Data Availability

The data that support the findings of this study are available on request from the corresponding author.
